# Postischemic Anhedonia Associated with Neurodegenerative Changes in the Hippocampal Dentate Gyrus of Rats

**DOI:** 10.1155/2016/5054275

**Published:** 2016-03-16

**Authors:** Jiro Kasahara, Hiroto Uchida, Kenta Tezuka, Nanae Oka

**Affiliations:** Department of Neurobiology and Therapeutics, Institute of Biomedical Sciences, Graduate School and Faculty of Pharmaceutical Sciences, Tokushima University, Tokushima 770-8505, Japan

## Abstract

Poststroke depression is one of the major symptoms observed in the chronic stage of brain stroke such as cerebral ischemia. Its pathophysiological mechanisms, however, are not well understood. Using the transient right middle cerebral artery occlusion- (MCAO-, 90 min) operated rats as an ischemia model in this study, we first observed that aggravation of anhedonia spontaneously occurred especially after 20 weeks of MCAO, and it was prevented by chronic antidepressants treatment (imipramine or fluvoxamine). The anhedonia specifically associated with loss of the granular neurons in the ipsilateral side of hippocampal dentate gyrus and was also prevented by an antidepressant imipramine. Immunohistochemical analysis showed increased apoptosis inside the granular cell layer prior to and associated with the neuronal loss, and imipramine seemed to recover the survival signal rather than suppressing the death signal to prevent neurons from apoptosis. Proliferation and development of the neural stem cells were increased transiently in the subgranular zone of both ipsi- and contralateral hippocampus within one week after MCAO and then decreased and almost ceased after 6 weeks of MCAO, while chronic imipramine treatment prevented them partially. Overall, our study suggests new insights for the mechanistic correlation between poststroke depression and the delayed neurodegenerative changes in the hippocampal dentate gyrus with effective use of antidepressants on them.

## 1. Introduction

Transient focal cerebral ischemia is the most common type of stroke caused by occlusion of a cerebral artery [1]. It causes both acute and chronic dysfunctions in the central nervous system (CNS) and lowers the quality of life in patients for a long period of lifetime. The middle cerebral artery (MCA) is most frequently infarcted in the cerebral ischemia, and various animal models have been developed including nonhuman primates and rodents [2, 3]. In the models of transient focal cerebral ischemia, neurons in the ischemic core including cerebral cortex and some parts of the striatum were immediately damaged after the ischemia-reperfusion manipulation, sometimes followed by the delayed neuronal death in the areas apart from the ischemic core, including a part of thalamus, substantia nigra, and hippocampus [4–6]. They cause various dysfunctions such as cognitive, mood/emotional, and motor impairments in the chronic stage after stroke.

Among the CNS dysfunctions in the chronic stage of cerebral ischemia, depression is one of the major mood/emotional impairments known as poststroke depression (PSD). It has been generally recognized that PSD occurred in around 40% of the stroke patients [7, 8], although it varies depending on the studies from around 20% [9] to 72% [10]. Because the physical disabilities lowering the activities of daily living are the stressor on the stroke patients, PSD has been believed to occur as the result of psychogenic and systemic responses to the stressed conditions with complicated mechanisms of pathogenesis [11]. Robinson and Price reported their follow-up study of 103 stroke patients with evaluating PSD [12, 13], confirming that the lesion location (frontal area in the left hemisphere and posterior area in the right hemisphere) determined frequency and severity of depression. It was the first study suggesting the PSD pathogenesis as a neurodegenerative lesion in a particular brain area. Now, PSD pathogenesis is considered to be multifactorial with neurodegenerative, psychogenic, and genetic mechanisms. Animal models of PSD have been reported especially using rodents [14], mostly combining a surgical operation (MCA occlusion) with application of extra stressors such as unpredicted chronic mild stress (UCMS) [15, 16], ovariectomy [17], and spatial restraint stress [18].

Among various symptoms seen in PSD, anhedonia is one of the typical ones: loss of interest or pleasure in almost all the activities and things that one previously liked [19]. Pathogenesis of anhedonia includes brain areas such as orbitofrontal cortex, nuclear accumbens, and ventral pallidum. A recent study reported a positive correlation between the postischemic anhedonia with salivary cortisol levels and reduction of volume by lesion in parahippocampal/hippocampal area [20].

Hippocampus is one of the vulnerable areas to the ischemic stress, showing delayed neuronal death in CA1 region within a few days to a week after MCA occlusion (MCAO) [6, 21, 22]. Because hippocampus is deeply related to higher brain functions such as cognition, learning, and memory, CA1 degeneration causes functional impairments of them after stroke. Different from CA1, other regions such as CA3 and dentate gyrus (DG) in the hippocampus are considered to be resistant to the ischemic stress [23, 24]. The neurogenesis in subgranular zone (SGZ) of DG as well as cortical subventricular zone (SVZ) produces newly generated neurons even in adulthood and is known to increase neural stem cells (NSCs) proliferation and differentiation into neurons after the transient brain ischemia [25, 26]. Because the rats that received UCMS after MCAO treatment had reduced neurogenesis in DG, it was considered as an adaptive or a compensatory process against the poststroke stressors [15]. Proliferation and differentiation of NSCs in SGZ are controlled by various factors such as stress, mood/emotion, environment, corticosteroids, and antidepressants [27, 28].

Based on these backgrounds, we initially examined whether anhedonia could be spontaneously induced after MCAO in rats while observing them for up to 30 weeks together with the effects of antidepressant imipramine (IMP) or fluvoxamine (FLV) in this study. The reason why we chose anhedonia rather than other depression-related behaviors such as the forced swimming or tail suspension was to minimize the negative effects of the motor impairments directly influenced by MCAO. We also evaluated the long-term neurodegenerative changes of the hippocampal DG, including apoptosis of the granular cells (GC) and the neurogenesis in SGZ, with the effects of IMP.

## 2. Materials and Methods

### 2.1. Experimental Animal

Young male SD rats weighing 200–250 g at 6 weeks of age were purchased from Nihon SLC, Co., Shizuoka, Japan. They were housed in a controlled environment (23 ± 1°C, 50 ± 5% humidity) and were allowed food and tap water* ad libitum* and habituated for 2 weeks before the surgical operation. The room lights were on between 8:00 and 20:00 (illuminated with 950 lux). The procedure of the surgical operations for transient MCAO was described in our previous publications [22, 29–31]. In brief, the right middle cerebral artery of the rat was occluded for 90 min by inserting a silicone-coated 4-0 monofilament after the right carotid artery was exposed and separated into the internal and the external ones with careful conservation of the vagus nerve under anesthetization with 2% halothane in a mixture of 30% oxygen and 70% nitrous oxide. One day after the reperfusion of blood flow by withdrawing the filament, animals that showed grade 3 or over of Hunter's neurological score [32] were used for further experiments. To examine the behavioral changes after the surgical operation for 30 weeks, 40 rats were divided into 4 groups: sham, sham + antidepressants, MCAO, and MCAO + antidepressants (*N* = 10/group, total *N* = 40). For the immunohistochemical examinations, another 80 rats were divided equally into two groups of sham and MCAO, and 5 rats/group were sacrificed at 1 day (1 d), 3 days (3 d), 1 week (1 w), 2 weeks (2 w), 6 weeks (6 w), 20 weeks (20 w), and 30 weeks (30 w) after the surgical operation. Naïve animal (*N* = 5) means the rats fixed without any surgical operation at 8 weeks of age.

All handling and procedures of animal experiments were performed in accordance with the National Institutes of Health* Guide for the Care and Use of Laboratory Animals* (NIH Publications number 8023, revised 1978) and approved by the Committee for Animal Experiments of Tokushima University.

### 2.2. Antidepressant Treatment

From 1 week after MCAO, the antidepressant imipramine hydrochloride or a selective serotonin reuptake inhibitor (SSRI) fluvoxamine maleate (Sigma, St. Louis, MO, USA) was dissolved directly in tap water based on the daily amount of water consumption so that the rats could absorb the drugs with 20 mg/kg/day. The doses were determined based on the previous reports [33–36]. For drug administration, we avoided a direct handling procedure such as intraperitoneal injection to minimize any noxious stressor on rats after MCAO.

### 2.3. Sucrose Preference Test

This test is to evaluate anhedonia, one of indices of depressiveness [37]. For habituation during one week before MCAO, animals were exposed to water containing 1% sucrose twice for 2 successive days separated by a 2-day interval substituted by normal tap water. The tests were performed at 1 w, 2 w, 6 w, 20 w, and 30 w after MCAO during the dark period (20:00–8:00) with presenting two bottles of water simultaneously (one was normal while the other one contained 1% sucrose), and the percentage of sucrose preference was calculated as (sucrose water intake)/(total water intake).

### 2.4. Open Field Test

This test was performed to evaluate the spontaneous activity of the rats at 1 w, 2 w, 6 w, 20 w, and 30 w after MCAO. The rats were put in an open field (50 cm × 50 cm, divided into 25 squares of 10 cm × 10 cm by line grids) under the 950-lux illumination for 10 min and were recorded by a video camera set at 60 cm high above the field. The number of stepping instances over the grids was counted as the horizontal activity, and the number of rearing instances was counted as the vertical activity, by an examiner unaware of the grouping details.

### 2.5. Immunohistochemistry

The procedure of the immunohistochemical examinations was followed essentially by the previous studies in our laboratory [22, 29–31]. Rats were anesthetized with intraperitoneal injection of 50 mg/kg of sodium pentobarbital solution at 1 d, 3 d, 1 w, 2 w, 6 w, 20 w, and 30 w after MCAO, flashed with 1% heparin-containing saline, and fixed with perfusion of 4% paraformaldehyde (Wako, Osaka, Japan) in phosphate buffered saline (PBS, pH 7.4, 137 mM NaCl, 2.7 mM KCl, 10 mM Na_2_HPO_4_, and 10 mM KH_2_PO_4_). One day after the fixation, brains were excised out and kept in the same fixative solution for one night. The fixed brains were washed with PBS for 1 hr × 3 times and dehydrated in ethanol (70, 80, 95, and 100%) and then embedded in paraffin. The coronal sections of the dorsal hippocampus (between bregmata −2.5 mm and −4.5 mm) with 5 *μ*m thickness were sliced out by a sliding microtome (TU-213, Yamato, Saitama, Japan), and 4 slices/animal (for one primary antibody) were taken out from each of the 0.5 mm ranges and mounted on the slide glasses. For deparaffinization, the slices were heated at 59°C for 1 hr, treated with xylene, ethanol, and dH_2_O, and then served for the immunohistochemical examinations. They were washed with PBS for 3 min × 3 times and incubated with the blocking solution containing 10% normal horse serum (Vector Labs, CA, USA) and 0.3% Triton X-100 in PBS for 60 min at room temperature.

For the primary antibodies of immunostaining, anti-neuronal nuclei (NeuN, monoclonal, Chemicon-Millipore, CA, USA, 1 : 200), anti-*β*III-tubulin (monoclonal, Abcam, MA, USA, 1 : 400), anti-MAP2 (monoclonal, Abcam, MA, USA, 1 : 1500), anti-Bax (polyclonal, Santa Cruz, CA, USA, 1 : 100), anti-Bcl-2*α* (monoclonal, Lab Vision, ND, USA, 1 : 50), anti-Nestin (monoclonal, Chemicon, CA, USA, 1 : 500), anti-Ki67 (polyclonal, Abcam, MA, USA, 1 : 400), and anti-doublecortin (DCX, polyclonal, Abcam, MA, USA, 1 : 1000) were used. They were diluted in the blocking solution and incubated with the slices for one night at 4°C. The slices were washed with PBS for 3 min × 3 times before incubating with the secondary antibody. To visualize the bound primary antibodies, a Vectastain elite ABC kit and 3′,3′-diaminobenzidine (DAB) substrate kit (Vector Labs, CA, USA) were used according to the manufacturer's protocol. As negative control sections, each primary antibody or the secondary antibody was omitted. All the slices on the slide glasses were covered by cover glasses with VECTASHIELD mounting medium (Vector Labs, CA, USA). They were observed and recorded with a digital camera-equipped BX51 light microscope (Olympus, Tokyo, Japan) at a magnitude of 12.5x (cresyl violet) or 200x (NeuN, Bax, Bcl-2*α*, and DCX) or 400x (*β*III-tubulin, MAP2, Nestin, and Ki67). To detect anti-cleaved caspase-3 antibody (polyclonal, Bio Vision, CA, USA, 1 : 100), TSA system with FITC kit (GE Health Science, MA, USA) combined with Höchst 33258 (Invitrogen, OR, USA) nuclear staining was used. The images were observed and captured with LSM510 confocal laser scanning microscope system (Carl Zeiss, Heidelberg, Germany) at a magnitude of 200x using ZEN 2009 interface with line mode, 3 *μ*m thickness of pinhole setting, and 4 × averaging with scan speed 4 (2 min, 5 sec).

### 2.6. Analysis of the Immunoreactivity

The images of DG from 4 slices/animal were captured and analyzed using a computer-associated image analyzer (WinROOF Version 5; Mitani Corporation, Fukui, Japan) by an examiner who was not aware of the grouping details. Images from one slice were collected from both ipsi- and contralateral DG so that 70% or more of the area of GCL could be examined. We defined the granular cell layer (GCL) in each image with 100 *μ*m width and SGZ at the inner edge of GCL with 20 *μ*m width and measured the defined area as mm^2^ automatically with the analyzer. The number of cresyl violet-stained or immunopositive cells (NeuN, cleaved caspase-3, Bax, Bcl-2*α*, Nestin, Ki67, and DCX) or % immunopositive density (*β*III-tubulin and MAP2) in each image was measured, summed up for 4 slices/animal, and was calculated as per mm^2^ of the measured area in each animal.

### 2.7. Statistical Analysis

All the data are given as means ± standard error of mean (SEM). The following analysis was used: behavioral examinations, two-way (time, group) analysis of variance (ANOVA) followed by Scheffe* post hoc* test at each time point; immunohistochemical examinations of the time course analysis, two-way (time, group) ANOVA followed by Tukey-Kramer* post hoc* test (Nissl, NeuN, *β*III-tubulin, MAP2, Nestin, Ki67, and DCX) or unpaired *t*-test (cleaved caspase-3, Bax, and Bcl-2*α*) at each time point; immunohistochemical examinations with IMP at 30 weeks after the surgical operation, one-way (group) ANOVA followed by Tukey-Kramer* post hoc* test; comparisons of two independent groups, unpaired *t*-test. *P* < 0.05 was regarded as statistically significant. All of the analysis was done using StatView Ver. 5.0 (SAS Institute, USA).

## 3. Results

### 3.1. Aggravation of Anhedonia after MCAO

**Figure 1:**
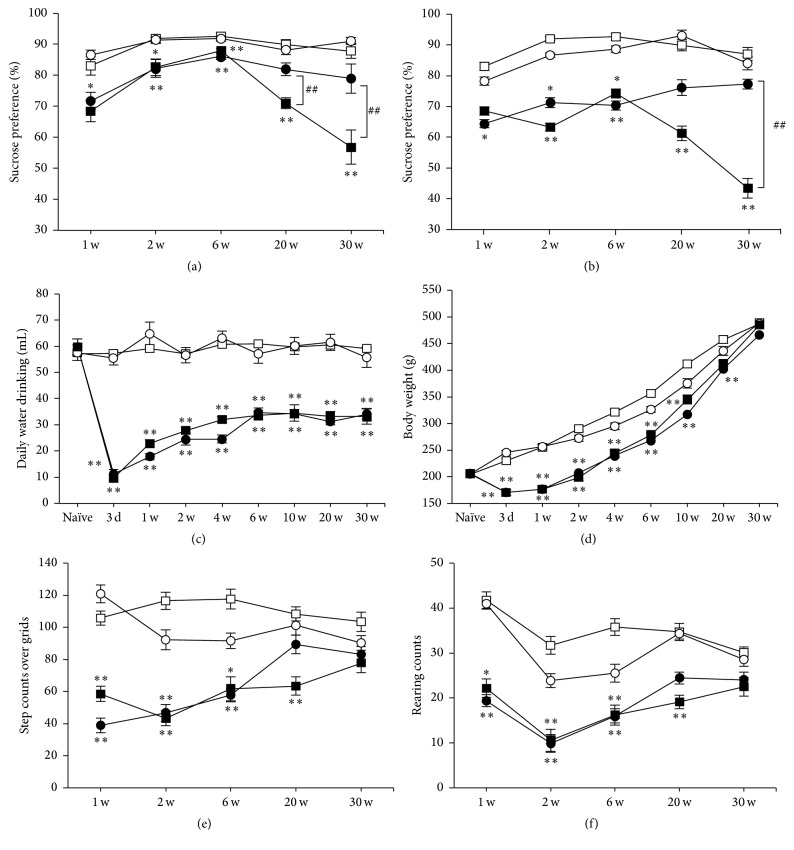
Behavioral analysis of the rats after MCAO. Symbols used are as follows: open square, sham; open circle, sham + antidepressant; closed square, MCAO; closed circle, MCAO + antidepressant; *N* = 10/group, total *N* = 40. For antidepressants treatment, vehicle (open and closed square), 20 mg/kg/day of IMP in (a) and (c) to (f) or FLV in (b) (open and closed circles) was administered for 29 weeks. Statistical significance was evaluated with two-way ANOVA followed by Scheffe* post hoc* test. (a) Sucrose preference test with IMP. *F*_(time) 4_ = 17.4; *F*_(group) 3_ = 63.9; *F*_(time*∗*group) 12,180_ = 9.131; ^*∗*^ *P* < 0.05 and ^*∗∗*^ *P* < 0.01 versus sham; ^##^ *P* < 0.01 versus MCAO + IMP. (b) Sucrose preference test with FLV. *F*_(time) 4_ = 2.76; *F*_(group) 3_ = 39.5; *F*_(time*∗*group) 12,180_ = 2.13; ^*∗*^ *P* < 0.05 and ^*∗∗*^ *P* < 0.01 versus sham; ^##^ *P* < 0.01 versus MCAO + FLV. (c) Daily amount of water drinking before (Naïve) and after MCAO. *F*_(time) 8_ = 27.2; *F*_(group) 3_ = 351.1; *F*_(time*∗*group) 24,324_ = 9.70; ^*∗∗*^ *P* < 0.01 versus sham. (d) Changes of body weight before (Naïve) and after MCAO. *F*_(time) 8_ = 1291.6; *F*_(group) 3_ = 249.5; *F*_(time*∗*group) 24,324_ = 11.1; ^*∗∗*^ *P* < 0.01 versus sham. (e, f) Open field test. (e) Horizontal activity during a period of 10 min. *F*_(time) 4_ = 3.43; *F*_(group) 3_ = 56.8; *F*_(time*∗*group) 12,180_ = 3.45; ^*∗∗*^ *P* < 0.01 versus sham. (f) Vertical activity characterized by the number of rearing instances during a period of 10 min. *F*_(time) 4_ = 9.79; *F*_(group) 3_ = 41.0; *F*_(time*∗*group) 12,180_ = 1.59; ^*∗∗*^ *P* < 0.01 versus sham.

We first examined whether depression-related behavior anhedonia would be spontaneously expressed after MCAO in rats. Normal rats usually show 80–90% of sucrose preference (87.9 ± 2.8% at 30 weeks in sham), as shown in Figures 1(a) and 1(b) (open symbols). MCAO-operated rats (closed symbols) tended to less prefer sucrose containing water compared to sham groups (open symbols) throughout the time course after MCAO. Interestingly, sucrose preference was further decreased significantly at 20 and 30 weeks after MCAO to around 50–60% (56.8 ± 4.5% at 30 weeks in MCAO, *P* < 0.01 versus sham), and it was prevented by chronic administration of IMP (Figure 1(a), 78.9 ± 3.8% at 30 weeks in MCAO + IMP, *P* < 0.05 versus MCAO) or FLV (Figure 1(b), 76.7 ± 2.2% in MCAO + FLV at 30 weeks, *P* < 0.05 versus MCAO) significantly. Daily amount of water consumption was decreased significantly to around 60% in MCAO-operated rats (59.2 ± 3.3 mL in sham versus 33.7 ± 1.8 mL in MCAO at 30 weeks, *P* < 0.01) and was not prevented by chronic IMP treatment (Figure 1(c), 34.4 ± 3.1 mL in MCAO + IMP at 30 weeks, *P* > 0.05 versus MCAO). The body weight of the MCAO-operated rats initially decreased significantly (230.1 ± 3.7 g in sham versus 170.4 ± 5.3 g in MCAO at 3 days, *P* < 0.01) and stayed lower than sham groups until around 6 weeks after MCAO and then gradually recovered and was almost the same as sham group at 30 weeks (Figure 1(d): 487.4 ± 6.2 g in sham versus 485.2 ± 7.1 g in MCAO at 30 weeks, *P* > 0.05). Chronic IMP treatment did not affect them after MCAO (465.9 ± 7.3 g in MCAO + IMP at 30 weeks, *P* > 0.05 versus MCAO). The rats given FLV also showed similar results to Figures 1(c) and 1(d) (data not shown).

### 3.2. Spontaneous Activity of the Rats after MCAO

We next examined the spontaneous activity of rats with open field test (Figures 1(e) and 1(f)). It revealed that both horizontal and vertical activities of MCAO-operated rats (closed symbols) were decreased significantly compared to sham groups (open symbols). However, they gradually recovered and reached a similar level to that of sham group at 30 weeks after MCAO (Figures 1(e) and 1(f): horizontal activity, 103.4 ± 4.3 in sham versus 77.6 ± 5.8 in MCAO, *P* > 0.05; vertical activity, 30.1 ± 1.2 in sham versus 23.5 ± 2.2 in MCAO, *P* > 0.05). Chronic IMP treatment did not affect them (Figures 1(e) and 1(f): horizontal activity, 83.2 ± 4.9 in MCAO + IMP, *P* > 0.05 versus MCAO; vertical activity, 24.2 ± 1.8 in MCAO + IMP, *P* > 0.05 versus MCAO). Similar results with FLV-treated rats were observed (data not shown).

### 3.3. Specific Loss of the Granular Neurons in the Ipsilateral DG after MCAO

**Figure 2:**
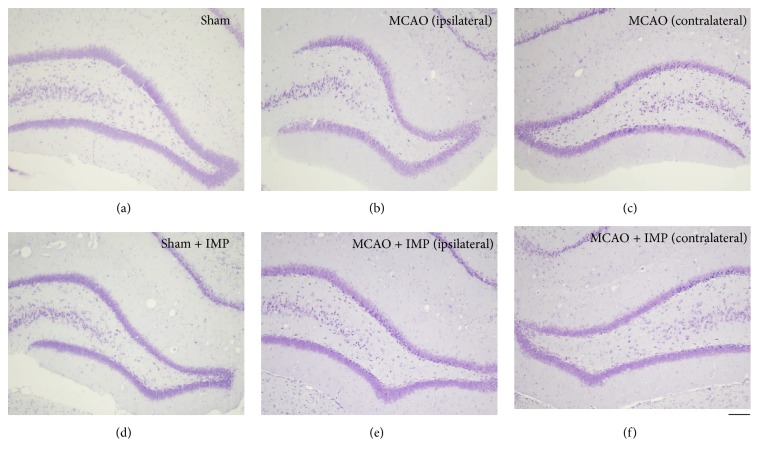
Typical images of the cresyl violet staining in the hippocampal dentate gyrus of rats. Note that the entire shape of GCL in (b) (ipsilateral MCAO) is shrunk compared to the others. Scale bar indicates 200 *μ*m.

**Figure 3:**
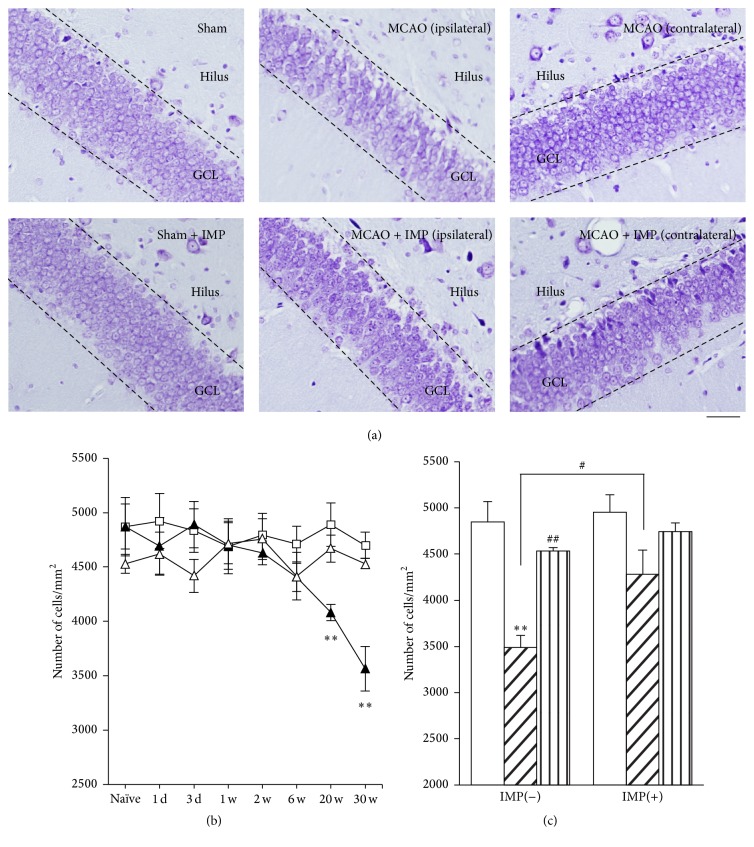
Analysis of the cresyl violet-stained cells in GCL. (a) Typical images of the slices taken at 30 weeks after MCAO. Scale bar indicates 50 *μ*m. (b) Time course changes of Nissl positive cells after MCAO in GCL. Symbols used are as follows: open square, sham; closed triangle, ipsilateral MCAO; open triangle, contralateral MCAO. Statistical significance was evaluated with two-way ANOVA followed by Tukey-Kramer test at each time point (*F*_(time) 7_ = 2.96; *F*_(group) 2_ = 7.37; *F*_(time*∗*group) 14,96_ = 2.42; ^*∗∗*^ *P* < 0.01 versus sham). (c) Analysis of the effect of IMP on the number of Nissl positive cells at 30 weeks after MCAO. Open columns, sham; hatched columns, ipsilateral MCAO; striped columns, contralateral MCAO. Vehicle [IMP(−)] or 20 mg/kg/day of IMP for 29 weeks [IMP(+)] from 1 week after the surgical operation was administered. Statistical significance was evaluated with one-way ANOVA followed by Tukey-Kramer test (*F*_5,24_ = 8.78; ^*∗∗*^ *P* < 0.01 versus sham [IMP(−)]; ^#^ *P* < 0.05 and ^##^ *P* < 0.01 versus ipsilateral MCAO; *N* = 5/group).

In the paraffin sections prepared from the brains at 30 weeks after MCAO, DG of the dorsal hippocampus was observed with cresyl violet staining (Figure 2). We noticed that the shape of GCL was shrunk specifically in the slices prepared from MCAO-operated ipsilateral hippocampus (Figure 2(b)). Time course analysis of the cresyl violet-stained cells revealed the significant loss of the cells in GCL observed at 20 and 30 weeks after MCAO (Figure 3(b): 4699.6 ± 119.2 cells/mm^2^ in sham versus 3561.8 ± 177.3 cells/mm^2^ in ipsilateral MCAO at 30 weeks, *P* < 0.01), and it was prevented by chronic administration of IMP from 1 week to 30 weeks after MCAO (Figure 3(c): 4531.0 ± 262.5 cells/mm^2^ in ipsilateral MCAO + IMP, *P* < 0.05 versus ipsilateral MCAO).

We also quantified the number of the cells in which the condensed cytosol was observed with cresyl violet staining at 30 weeks after MCAO (*F*_5,24_ = 1.67, one-way ANOVA followed by Tukey-Kramer* post hoc* test). Compared with sham (102.7 ± 14.7 cells/mm^2^), MCAO tended to increase the condensed cells in both ipsi- and contralateral GCL without statistical significance (147.5 ± 9.6 cells/mm^2^ in ipsilateral MCAO and 129.2 ± 16.7 cells/mm^2^ in contralateral MCAO, *P* > 0.05 versus sham), and IMP did not reverse them (137.4 ± 14.8 cells/mm^2^ in ipsilateral MCAO + IMP and 133.2 ± 21.4 cells/mm^2^ in contralateral MCAO + IMP, *P* > 0.05 versus sham).

**Figure 4:**
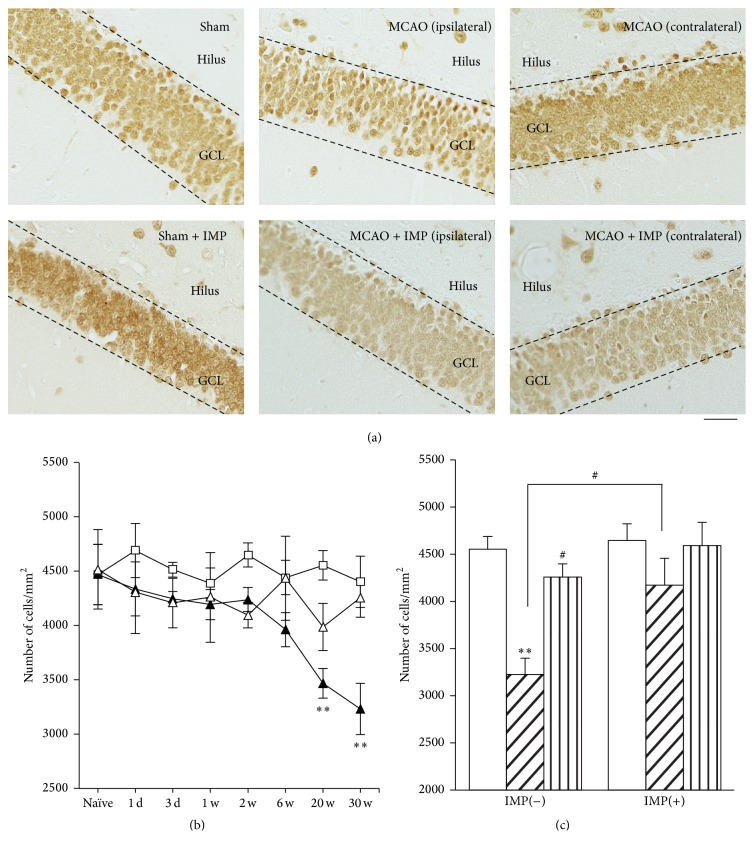
Analysis of NeuN immunopositive cells in GCL. (a) Typical images of the slices taken at 30 weeks after MCAO. Scale bar indicates 50 *μ*m. (b) Time course changes of NeuN immunopositive cells after MCAO in GCL. Symbols used are as follows: open square, sham; closed triangle, ipsilateral MCAO; open triangle, contralateral MCAO. Statistical significance was evaluated with two-way ANOVA followed by Tukey-Kramer test at each time point (*F*_(time) 7_ = 2.78; *F*_(group) 2_ = 11.0; *F*_(time*∗*group) 14,96_ = 1.57; ^*∗∗*^ *P* < 0.01 versus sham). (c) Analysis of the effect of IMP on the number of NeuN immunopositive cells at 30 weeks after MCAO. Vehicle [IMP(−)] or 20 mg/kg/day of IMP for 29 weeks [IMP(+)] from 1 week after the surgical operation was administered. Statistical significance was evaluated with one-way ANOVA followed by Tukey-Kramer test (*F*_5,24_ = 7.00; ^*∗∗*^ *P* < 0.01 versus sham [IMP(−)]; ^#^ *P* < 0.05 versus ipsilateral MCAO [IMP(−)]; *N* = 5/group).

Because loss of GC in DG after ischemia had never been reported yet, we examined NeuN immunoreactivity as a marker of the matured neurons in GCL (Figure 4). Similar to the result of cresyl violet staining in Figure 3, time course analysis revealed that the number of NeuN immunopositive cells in GCL was decreased at 20 and 30 weeks after MCAO significantly (Figure 4(b): 4401.0 ± 110.4 cells/mm^2^ in sham versus 3230.9 ± 235.8 cells/mm^2^ in ipsilateral MCAO at 30 weeks, *P* < 0.01) and the decrease was prevented by chronic IMP treatment for 29 weeks after MCAO (Figure 4(c): 4169.8 ± 287.5 cells/mm^2^ in ipsilateral MCAO + IMP, *P* < 0.05 versus ipsilateral MCAO).

**Figure 5:**
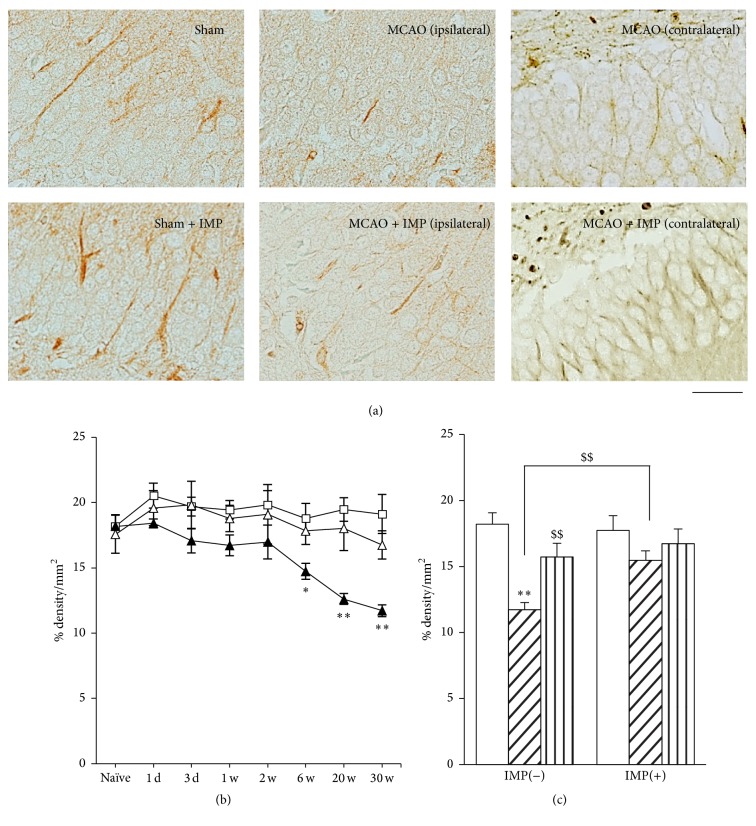
Analysis of *β*III-tubulin immunopositive areas in GCL. (a) Typical images of the slices taken at 30 weeks after MCAO. Scale bar indicates 30 *μ*m. (b) Time course changes of *β*III-tubulin immunopositive areas after MCAO in GCL. Symbols used are as follows: open square, sham; closed triangle, ipsilateral MCAO; open triangle, contralateral MCAO. Statistical significance was evaluated with two-way ANOVA followed by Tukey-Kramer test at each time point (*F*_(time) 7_ = 3.06; *F*_(group) 2_ = 19.6; *F*_(time*∗*group) 14,96_ = 1.17; ^*∗*^ *P* < 0.05 and ^*∗∗*^ *P* < 0.01 versus sham). (c) Analysis of the effect of IMP on *β*III-tubulin immunopositive areas at 30 weeks after MCAO. Vehicle [IMP(−)] or 20 mg/kg/day of IMP for 29 weeks [IMP(+)] from 1 week after the surgical operation was administered. Statistical significance was evaluated with one-way ANOVA followed by Tukey-Kramer test (*F*_5,24_ = 6.05; ^*∗∗*^ *P* < 0.01 versus sham [IMP(−)]; *N* = 5/group). Unpaired *t*-test was performed with ipsilateral MCAO [IMP(−)] versus contralateral MCAO [IMP(−)] (*t* = 3.41, ^$$^ *P* < 0.01) or versus ipsilateral MCAO [IMP(+)] (*t* = 4.12, ^$$^ *P* < 0.01).

**Figure 6:**
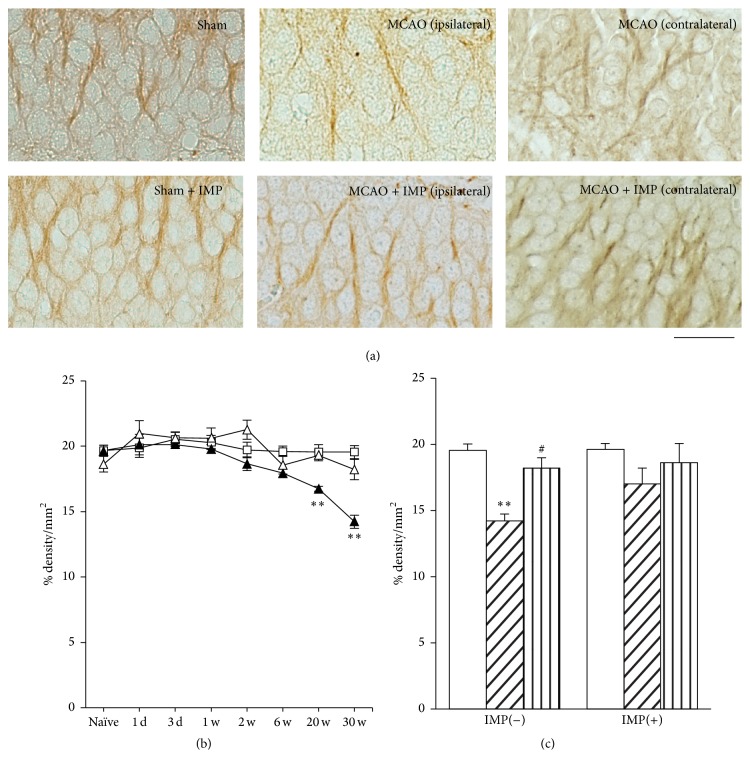
Analysis of MAP2 immunopositive areas in GCL. (a) Typical images of the slices taken at 30 weeks after MCAO. Scale bar indicates 20 *μ*m. (b) Time course changes of MAP2 immunopositive areas after MCAO in GCL. Symbols used are as follows: open square, sham; closed triangle, ipsilateral MCAO; open triangle, contralateral MCAO. Statistical significance was evaluated with two-way ANOVA followed by Tukey-Kramer test at each time point (*F*_(time) 7_ = 11.0; *F*_(group) 2_ = 16.1; *F*_(time*∗*group) 14,96_ = 3.65; ^*∗∗*^ *P* < 0.01 versus sham). (c) Analysis of the effect of IMP on MAP2 immunopositive areas at 30 weeks after MCAO. Vehicle [IMP(−)] or 20 mg/kg/day of IMP for 29 weeks [IMP(+)] from 1 week after the surgical operation was administered. Statistical significance was evaluated with one-way ANOVA followed by Tukey-Kramer test (*F*_5,24_ = 5.07; ^*∗∗*^ *P* < 0.01 versus sham [IMP(−)]; ^#^ *P* < 0.05 versus ipsilateral MCAO [IMP(−)]; *N* = 5/group).

We further examined other antibodies for the neuronal markers *β*III-tubulin and MAP2 (Figures 5 and 6) in GCL with measuring % density of the immunoreactive area. Immunoreactivity of *β*III-tubulin in ipsilateral GCL started to decrease at 6 weeks after MCAO (Figure 5(b): 18.8 ± 0.90% in sham versus 14.6 ± 0.61% in ipsilateral MCAO, *P* < 0.05) and further decreased at 20 and 30 weeks (12.4 ± 0.48%, *P* < 0.01 versus sham), and it was reversed by chronic IMP treatment (15.7 ± 0.72%, *P* < 0.01). Similar results were obtained from the examination of MAP2 immunoreactivity (Figure 6). At 30 weeks after the surgical operation, 19.6 ± 0.50% in sham was decreased to 14.2 ± 0.51% in ipsilateral MCAO (*P* < 0.01), and chronic IMP treatment prevented it (17.0 ± 1.20%, *P* > 0.05 versus sham).

All of these data indicated the degenerative loss of GC in the ipsilateral DG at chronic stages after MCAO.

### 3.4. Increased Apoptosis in GCL after MCAO

**Figure 7:**
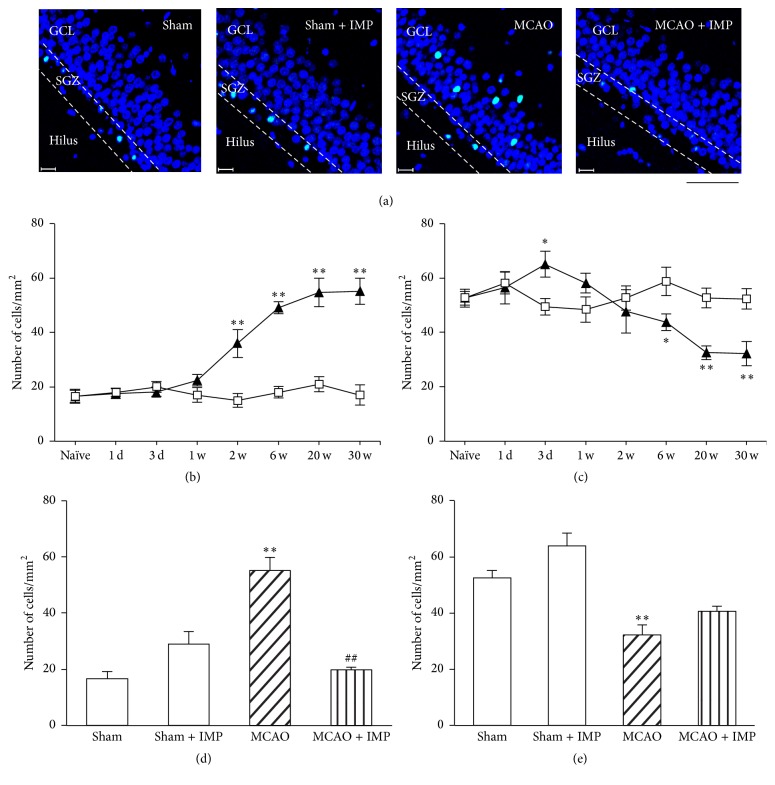
Analysis of the number of cleaved caspase-3 immunopositive cells in ipsilateral GCL and SGZ. (a) Typical images of the slices taken at 30 weeks after MCAO. Blue signal is the nuclei stained with Höchst 33258, while green signal of FITC is from cleaved caspase-3 immunopositive cells. Scale bar indicates 50 *μ*m. (b, c) Time course changes of cleaved caspase-3 immunopositive cells after MCAO in GCL (b) and SGZ (c). Symbols used are as follows: open square, sham; closed triangle, MCAO. Statistical significance was evaluated with two-way ANOVA followed by unpaired *t*-test at each time point (GCL: *F*_(time) 7_ = 21.2, *F*_(group) 1_ = 129.6, *F*_(time*∗*group) 7,64_ = 19.1, *t*_(2 w)_ = 3.71, *t*_(6 w)_ = 12.35, *t*_(20 w)_ = 6.24, and *t*_(30 w)_ = 7.53; SGZ: *F*_(time) 7_ = 4.50, *F*_(group) 1_ = 5.84, *F*_(time*∗*group) 7,64_ = 6.06, *t*_(3 d)_ = 3.29, *t*_(6 w)_ = −2.79, *t*_(20 w)_ = −4.86, and *t*_(30 w)_ = −3.95; ^*∗*^ *P* < 0.05 and ^*∗∗*^ *P* < 0.01 versus sham). (d, e) Analysis of the effect of IMP (20 mg/kg/day for 29 weeks) on the number of cleaved caspase-3 immunopositive cells at 30 weeks after MCAO in GCL (d) and SGZ (e). Statistical significance was evaluated with one-way ANOVA followed by Tukey-Kramer* post hoc* test (*F*_(GCL) 3,16_ = 24.63; *F*_(SGZ) 3,16_ = 18.41; ^*∗∗*^ *P* < 0.01 versus sham [IMP(−)]; ^##^ *P* < 0.01 versus MCAO [IMP(−)]; *N* = 5/group).

In Section 3.3, we showed that delayed neuronal loss occurred in the ipsilateral GCL of the hippocampal DG at chronic stages after MCAO. To examine whether the degenerative loss of GC involved neuronal apoptosis, we next performed immunostaining of cleaved caspase-3, which executes apoptosis. We analyzed the ipsilateral GCL and SGZ separately to distinguish the matured neurons and the other cells including NSCs in DG. In GCL, the number of cleaved caspase-3 immunopositive cells was significantly increased at 2 through 30 weeks of MCAO (Figure 7(b): 17.7 ± 1.44 cells/mm^2^ in sham versus 55.1 ± 4.76 cells/mm^2^ in MCAO at 30 weeks, *P* < 0.01) and the decrease was mostly prevented by chronic IMP treatment at 30 weeks after MCAO (Figures 7(a) and 7(d): 19.8 ± 1.01 cells/mm^2^ in MCAO + IMP at 30 weeks, *P* < 0.01 versus MCAO). In contrast, the number of cleaved caspase-3 immunopositive cells in SGZ was initially increased at 3 days (49.3 ± 3.03 cells/mm^2^ in sham versus 65.1 ± 3.71 cells/mm^2^ in MCAO, *P* < 0.05) and then decreased at 6 through 30 weeks of MCAO (Figure 7(c): 52.3 ± 3.73 cells/mm^2^ in sham versus 32.2 ± 3.48 cells/mm^2^ in MCAO at 30 weeks, *P* < 0.01), and chronic IMP treatment tended to prevent it (Figure 7(e): 40.6 ± 1.79 cells/mm^2^, *P* > 0.05 versus sham). It is notable that the number of cleaved caspase-3 immunopositive cells in SGZ was much higher than that of GCL in sham groups, whereas it was reversed after 20 and 30 weeks of MCAO, suggesting the increased apoptosis of the matured GC and decreased apoptosis of NSCs in DG after MCAO.

### 3.5. Analysis of the Death and Survival Signaling in DG after MCAO

**Figure 8:**
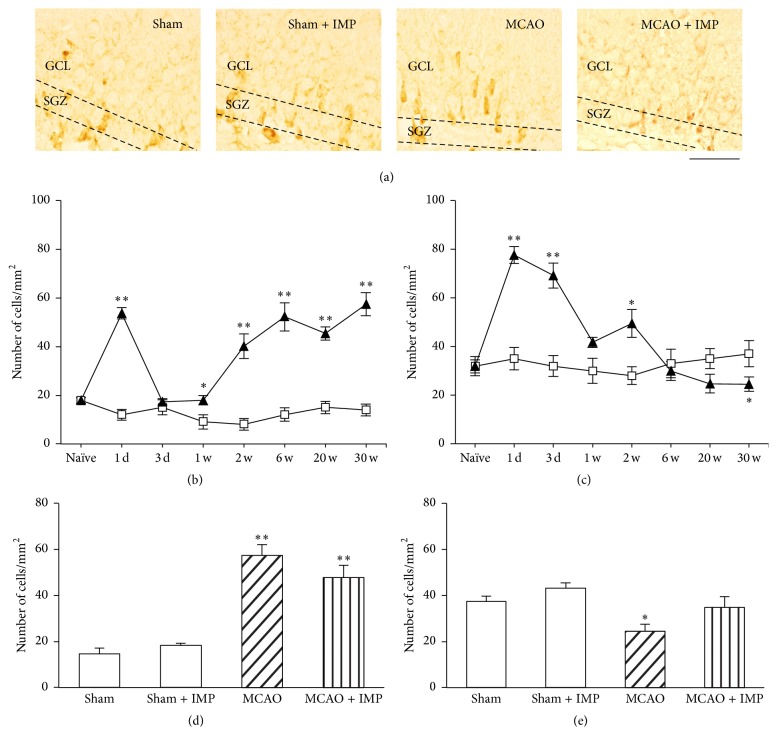
Analysis of the number of Bax immunopositive cells in the ipsilateral GCL and SGZ. (a) Typical images of the slices taken at 30 weeks after MCAO. Scale bar indicates 50 *μ*m. (b, c) Time course changes of Bax immunopositive cells after MCAO in GCL (b) and SGZ (c). Symbols used are as follows: open square, sham; closed triangle, MCAO. Statistical significance was evaluated with two-way ANOVA followed by unpaired *t*-test at each time point (GCL: *F*_(time) 7_ = 18.1, *F*_(group) 1_ = 286.4, *F*_(time*∗*group) 7,64_ = 19.3, *t*_(1 d)_ = 12.8, *t*_(1 w)_ = 2.46, *t*_(2 w)_ = 5.61, *t*_(6 w)_ = 6.30, *t*_(20 w)_ = 11.3, and *t*_(30 w)_ = 7.95; SGZ: *F*_(time) 7_ = 11.2, *F*_(group) 1_ = 27.8, *F*_(time*∗*group) 7,64_ = 12.3, *t*_(1 d)_ = 7.51, *t*_(3 d)_ = 5.52, *t*_(2 w)_ = 3.17, and *t*_(30 w)_ = −2.00; ^*∗*^ *P* < 0.05 and ^*∗∗*^ *P* < 0.01 versus sham). (d, e) Analysis of the effect of IMP (20 mg/kg/day for 29 weeks) on the number of Bax immunopositive cells at 30 weeks after MCAO in GCL (d) and SGZ (e). Statistical significance was evaluated with one-way ANOVA followed by Tukey-Kramer* post hoc* test (*F*_(GCL) 3,16_ = 31.1; *F*_(SGZ) 3,16_ = 6.07; ^*∗*^ *P* < 0.05 and ^*∗∗*^ *P* < 0.01 versus sham [IMP(−)]; *N* = 5/group).

**Figure 9:**
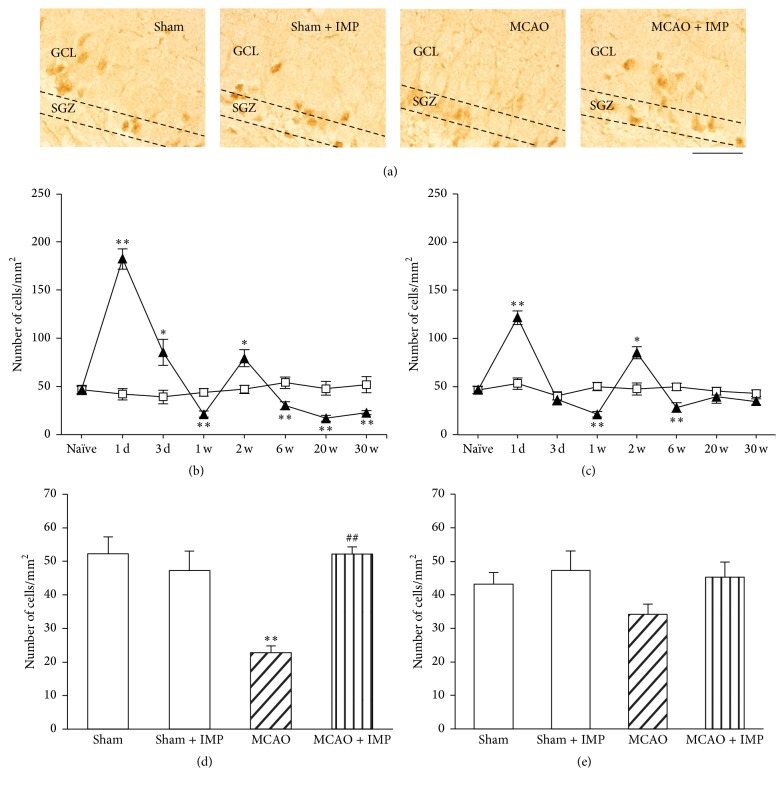
Analysis of the number of Bcl-2*α* immunopositive cells in the ipsilateral GCL and SGZ. (a) Typical images of the slices taken at 30 weeks after MCAO. Scale bar indicates 50 *μ*m. (b, c) Time course changes of Bcl-2*α* immunopositive cells after MCAO in GCL (b) and SGZ (c). Symbols used are as follows: open square, sham; closed triangle, MCAO. Statistical significance was evaluated with two-way ANOVA followed by unpaired *t*-test at each time point (GCL: *F*_(time) 7_ = 40.3, *F*_(group) 1_ = 20.8, *F*_(time*∗*group) 7,64_ = 48.7, *t*_(1 d)_ = 12.4, *t*_(3 d)_ = 3.15, *t*_(1 w)_ = −5.54, *t*_(2 w)_ = 3.31, *t*_(6 w)_ = −4.16, *t*_(20 w)_ = −5.73, and *t*_(30 w)_ = −5.44; SGZ: *F*_(time) 7_ = 23.1, *F*_(group) 1_ = 2.36, *F*_(time*∗*group) 7,64_ = 18.2, *t*_(1 d)_ = 7.36, *t*_(1 w)_ = −5.38, *t*_(2 w)_ = 2.90, and *t*_(6 w)_ = −3.64; ^*∗*^ *P* < 0.05 and ^*∗∗*^ *P* < 0.01 versus sham). (d, e) Analysis of the effect of IMP (20 mg/kg/day for 29 weeks) on the number of Bcl-2*α* immunopositive cells at 30 weeks after MCAO in GCL (d) and SGZ (e). Statistical significance was evaluated with one-way ANOVA followed by Tukey-Kramer* post hoc* test (*F*_(GCL) 3,16_ = 11.9; *F*_(SGZ) 3,16_ = 1.84; ^*∗∗*^ *P* < 0.01 versus sham [IMP(−)]; ^##^ *P* < 0.01 versus MCAO; *N* = 5/group).

Related to the increased apoptosis in the ipsilateral GCL, we characterized the immunoreactivities of Bax as a marker of death signaling and Bcl-2*α* as a marker of survival signaling in DG using specific antibodies for them (Figures 8 and 9, Supplementary Figures 1 and 2 in Supplementary Material available online at http://dx.doi.org/10.1155/2016/5054275).

In the ipsilateral GCL, Bax immunoreactivity increased transiently at 1 day after MCAO (Figure 8(b): 11.9 ± 2.23 cells/mm^2^ in sham versus 53.7 ± 2.38 cells/mm^2^ in MCAO, *P* < 0.01), returned to the sham level in 3 days, and then increased again and stayed higher than the sham group significantly at 1 through 30 weeks of MCAO (Figure 8(b): 14.7 ± 2.47 cells/mm^2^ in sham versus 57.4 ± 4.78 cells/mm^2^ in MCAO at 30 weeks, *P* < 0.01). The increased Bax was not prevented by chronic IMP treatment at 30 weeks after MCAO (Figure 8(d): 47.8 ± 5.29 cells/mm^2^ in MCAO + IMP, *P* < 0.01 versus sham). In the ipsilateral SGZ, Bax immunoreactivity showed a biphasic increase with its initial peak at 1–3 days (Figure 8(c): 77.7 ± 3.52 cells/mm^2^ at 1 day, *P* < 0.01) and the second peak at 2 weeks (49.4 ± 5.70 cells/mm^2^, *P* < 0.05) and then returned to the sham level and was lower than sham at 30 weeks after MCAO (24.5 ± 2.97 cells/mm^2^, *P* < 0.05). Chronic IMP treatment tended to prevent this decrease at 30 weeks (Figure 8(e): 34.8 ± 4.66 cells/mm^2^, *P* > 0.05 versus sham). The same analysis was performed in the contralateral DG (Supplementary Figure 1). Different from the ipsilateral GCL, Bax immunoreactivity in the contralateral GCL only showed the initial increase at 1 day (Supplementary Figure 1B: 16.7 ± 2.98 cells/mm^2^ in sham versus 61.9 ± 5.38 cells/mm^2^ in MCAO, *P* < 0.01) and returned to the basal level at 3 days through 30 weeks after MCAO, and chronic IMP treatment had no effect on it (Supplementary Figure 1D). The contralateral SGZ showed a similar time course change to the ipsilateral SGZ, although it only had a significant increase at 1 and 3 days after MCAO (Supplementary Figure 1C: 28.5 ± 4.31 cells/mm^2^ in sham versus 73.31 ± 3.80 cells/mm^2^ in MCAO at 1 day, *P* < 0.01) and returned to the sham level at 1 week through 30 weeks after MCAO. Chronic IMP treatment had no effect on it (Supplementary Figure 1E).

Immunoreactivity of Bcl-2*α* in the ipsilateral GCL (Figure 9(b): 47.0 ± 1.50 cells/mm^2^ in sham throughout the time course) showed a biphasic increase with two peaks at 1 day (182.4 ± 10.7 cells/mm^2^, *P* < 0.01) and 2 weeks (79.4 ± 8.99 cells/mm^2^, *P* < 0.05) after MCAO. However, it was significantly lower than sham at 1, 6, 20, and 30 weeks (22.8 ± 2.04 cells/mm^2^ at 30 weeks, *P* < 0.01), and chronic IMP treatment normalized it at 30 weeks (Figure 9(d): 52.2 ± 2.15 cells/mm^2^, *P* < 0.05 versus MCAO). Similar time course change of Bcl-2*α* to GCL was observed in the ipsilateral SGZ with two peaks at 1 day (121.6 ± 7.11 cells/mm^2^, *P* < 0.01) and 2 weeks (85.2 ± 6.38 cells/mm^2^, *P* < 0.05) after MCAO (Figure 9(c)). As was in GCL, it was significantly lower than sham at 1 and 6 weeks, although it returned to the sham level at 20 and 30 weeks after MCAO, and there was no difference between sham and MCAO groups with or without IMP (Figure 9(e)). In the contralateral GCL (Supplementary Figure 2B: 45.9 ± 1.68 cells/mm^2^ throughout the time course in sham), it also showed a biphasic increase with two peaks at 1 day (268.1 ± 6.60 cells/mm^2^, *P* < 0.01) and 2 weeks (139.3 ± 25.2 cells/mm^2^, *P* < 0.01). Different from the ipsilateral GCL, it never dropped below the sham level throughout the time course. At 30 weeks after MCAO, there was no significant difference between sham and MCAO with or without chronic IMP treatment (Supplementary Figure 2D). Similar result was obtained from the contralateral SGZ (Supplementary Figures 2C and 2E).

**Figure 10:**
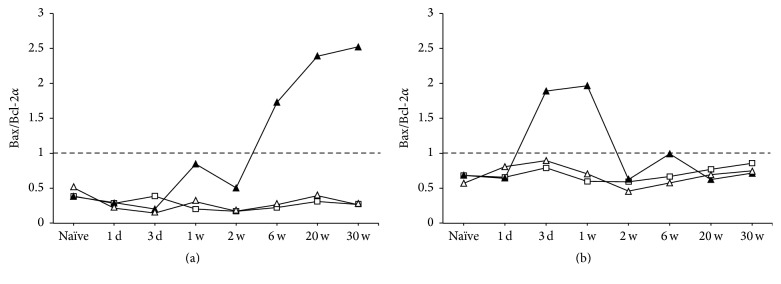
Time course changes of Bax/Bcl-2*α* index in GCL (a) and SGZ (b). Symbols used are as follows: open square, sham; closed triangles, ipsilateral MCAO; open triangles, contralateral MCAO.

From these results of Bax and Bcl-2*α*, we calculated Bax/Bcl-2*α* ratio [38] to see how the death and the survival signaling were changed after MCAO in GCL and in SGZ (Figure 10). In the ipsilateral GCL (Figure 10(a), closed triangle), it exceeded 1.0 at 6 through 30 weeks after MCAO, suggesting that the death signaling was dominant in chronic stages. In contrast, the index in SGZ (Figure 10(b)) increased at early stages after MCAO, exceeded 1.0 at 3 days and 1 week, and returned to the normal level at 2 through 30 weeks after MCAO. In the contralateral GCL and SGZ (open triangle), it kept below 1.0 as was in sham (open square) throughout the time course for 30 weeks after MCAO.

### 3.6. Analysis of Neural Stem Cell Proliferation and Development in SGZ after MCAO

We further analyzed the proliferation and development of NSCs in SGZ because they are well related to mood, depressiveness, and effects of antidepressants.

**Figure 11:**
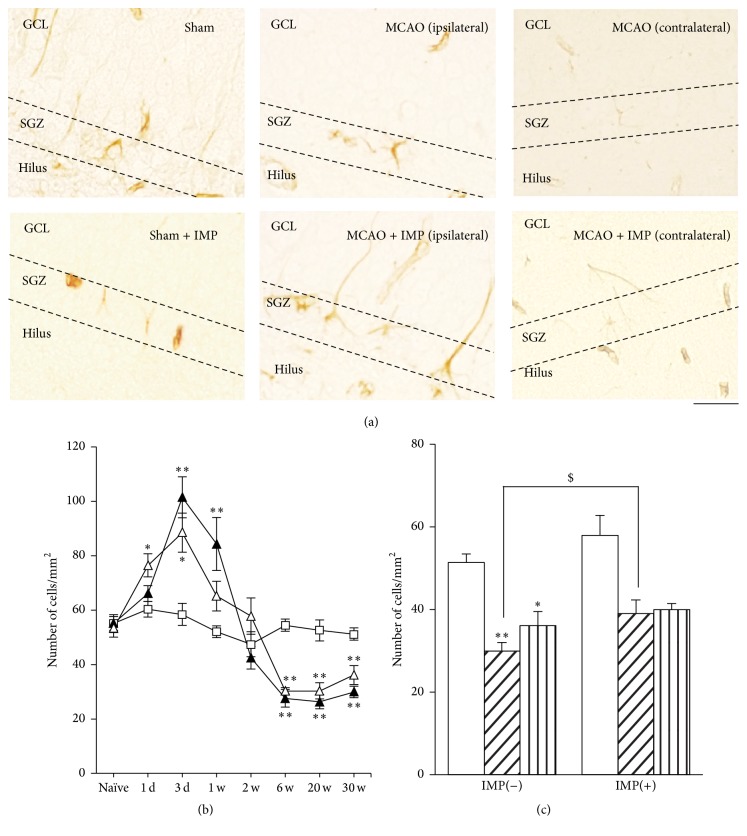
Analysis of Nestin immunopositive cells in GCL. (a) Typical images of the slices taken at 30 weeks after MCAO. Scale bar indicates 20 *μ*m. (b) Time course changes of Nestin immunopositive cells after MCAO in GCL. Symbols used are as follows: open square, sham; closed triangle, ipsilateral MCAO; open triangle, contralateral MCAO. Statistical significance was evaluated with two-way ANOVA followed by Tukey-Kramer test at each time point (*F*_(time) 7_ = 53.9; *F*_(group) 2_ = 0.097;  *F*_(time*∗*group) 14,96_ = 12.5; ^*∗*^ *P* < 0.05 and ^*∗∗*^ *P* < 0.01 versus sham). (c) Analysis of the effect of imipramine (IMP) on the number of Nestin immunopositive cells at 30 weeks after MCAO. Vehicle [IMP(−)] or 20 mg/kg/day of IMP for 29 weeks [IMP(+)] from 1 week after the surgical operation was administered. Statistical significance was evaluated with one-way ANOVA followed by Tukey-Kramer test (*F*_5,24_ = 11.36; ^*∗*^ *P* < 0.05 and ^*∗∗*^ *P* < 0.01 versus sham [IMP(−)]; *N* = 5/group). Unpaired *t*-test was performed with ipsilateral MCAO [IMP(−)] versus [IMP(+)] (*t* = −2.40, ^$^ *P* < 0.05).

In MCAO rats, immunoreactivity of NSCs marker Nestin increased transiently at 3 days and 1 week after MCAO in both the ipsilateral and contralateral SGZ of DG (Figure 11(b) at 3 days: 58.4 ± 4.13 cells/mm^2^ in sham; 101.4 ± 7.55 cells/mm^2^ in ipsilateral SGZ, *P* < 0.01 versus sham; 88.4 ± 7.18 cells/mm^2^ in contralateral SGZ, *P* < 0.05 versus sham) and then decreased and reached almost 50% of sham group (at 30 weeks: 29.9 ± 2.12 cells/mm^2^ in ipsilateral MCAO, *P* < 0.01 versus sham; 36.1 ± 3.5 cells/mm^2^ in contralateral MCAO, *P* < 0.01 versus sham). Chronic IMP treatment for 29 weeks significantly prevented the decrease of Nestin-positive cells observed in ipsilateral MCAO group at 30 weeks (39.9 ± 1.47 cells/mm^2^, *P* < 0.05 versus MCAO), although they were still lower than those of sham group significantly (Figure 11(c)).

**Figure 12:**
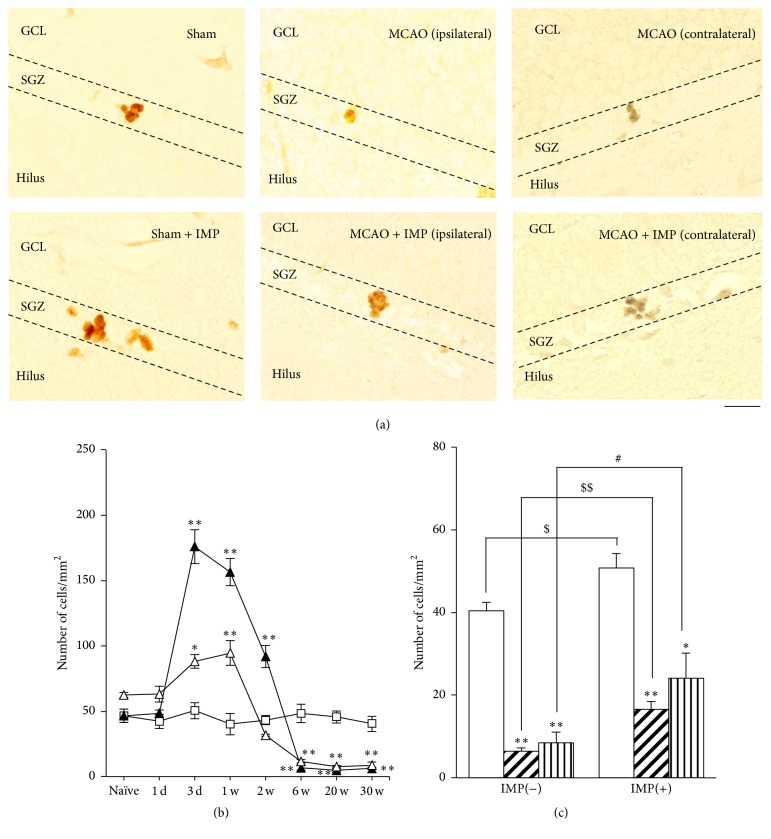
Analysis of Ki67 immunopositive cells in GCL. (a) Typical images of the slices taken at 30 weeks after MCAO. Scale bar indicates 20 *μ*m. (b) Time course changes of Ki67 immunopositive cells after MCAO in GCL. Symbols used are as follows: open square, sham; closed triangle, ipsilateral MCAO; open triangle, contralateral MCAO. Statistical significance was evaluated with two-way ANOVA followed by Tukey-Kramer test at each time point (*F*_(time) 7_ = 101.1; *F*_(group) 2_ = 38.2; *F*_(time*∗*group) 14,96_ = 37.7; ^*∗∗*^ *P* < 0.01 versus sham). (c) Analysis of the effect of imipramine (IMP) on the number of Ki67 immunopositive cells at 30 weeks after MCAO. Vehicle [IMP(−)] or 20 mg/kg/day of IMP for 29 weeks [IMP(+)] from 1 week after the surgical operation was administered. Statistical significance was evaluated with one-way ANOVA followed by Tukey-Kramer test (*F*_5,24_ = 29.53; ^*∗*^ *P* < 0.05 and ^*∗∗*^ *P* < 0.01 versus sham [IMP(−)]; ^#^ *P* < 0.05 versus contralateral MCAO [IMP(−)]; *N* = 5/group). Unpaired *t*-test was performed with sham [IMP(−)] versus [IMP(+)] (*t* = −2.53, ^$^ *P* < 0.05) or with ipsilateral MCAO [IMP(−)] versus [IMP(+)] (*t* = −4.74, ^$$^ *P* < 0.01).

We also examined Ki67 immunoreactivity as a proliferating cell marker. The result was similar to those observed in Nestin. Ki67 immunoreactivity increased transiently with its peak at 3 days after MCAO (Figure 12(b) at 3 days: 50.4 ± 6.29 cells/mm^2^ in sham; 175.9 ± 12.8 cells/mm^2^ in ipsilateral SGZ, *P* < 0.01 versus sham; 90.1 ± 5.12 cells/mm^2^ in contralateral SGZ, *P* < 0.05 versus sham) and then decreased and stayed with a few cells per mm^2^ at 6 through 30 weeks (Figure 12(b) at 30 weeks: 6.40 ± 0.79 cells/mm^2^ in ipsilateral MCAO, *P* < 0.01 versus sham; 8.51 ± 2.55 cells/mm^2^ in contralateral MCAO, *P* < 0.01 versus sham). Chronic IMP treatment significantly prevented the decreased Ki67 immunoreactivity at 30 weeks after MCAO in all groups, although they were still around 50% of sham group (Figure 12(c): 16.5 ± 1.98 cells/mm^2^ in ipsilateral MCAO + IMP; 24.1 ± 6.05 cells/mm^2^ in contralateral MCAO + IMP; *P* < 0.05 versus its corresponding MCAO).

**Figure 13:**
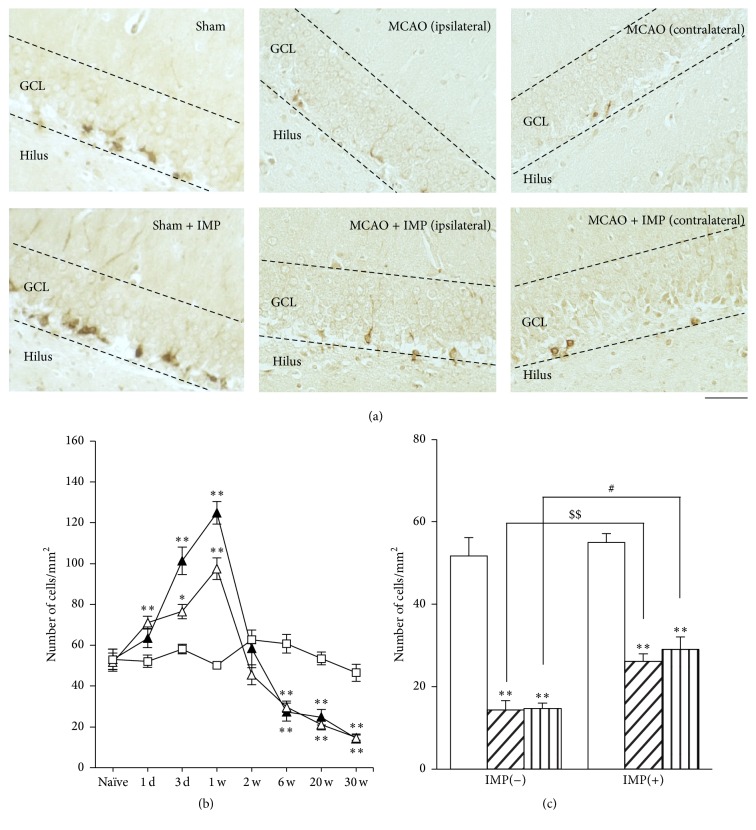
Analysis of DCX immunopositive cells in GCL. (a) Typical images of the slices taken at 30 weeks after MCAO. Scale bar indicates 50 *μ*m. (b) Time course changes of DCX immunopositive cells after MCAO in GCL. Symbols used are as follows: open square, sham; closed triangle, ipsilateral MCAO; open triangle, contralateral MCAO. Statistical significance was evaluated with one-way ANOVA followed by Tukey-Kramer test at each time point (*F*_(time) 7_ = 80.7; *F*_(group) 2_ = 5.91; *F*_(time*∗*group) 14,96_ = 22.7; ^*∗∗*^ *P* < 0.01 versus sham; ^#^ *P* < 0.05 versus contralateral MCAO [IMP(−)]; *N* = 5/group). (c) Analysis of the effect of imipramine (IMP) on the number of DCX immunopositive cells at 30 weeks after MCAO. Vehicle [IMP(−)] or 20 mg/kg/day of IMP for 29 weeks [IMP(+)] from 1 week after the surgical operation was administered. Statistical significance was evaluated with one-way ANOVA followed by Tukey-Kramer test (*F*_5,24_ = 41.60; ^*∗∗*^ *P* < 0.01 versus sham; ^#^ *P* < 0.05 versus contralateral MCAO [IMP(−)]; *N* = 5/group). Unpaired *t*-test was performed with ipsilateral MCAO [IMP(−)] versus [IMP(+)] (*t* = −4.10, ^$$^ *P* < 0.01).

Finally, we examined DCX immunoreactivity as a developmental marker of neurons in GCL. Although we analyzed entire GCL, most of the immunopositive cells are found around SGZ. As was observed in Nestin and Ki67, it also increased in early stages with its peak at 1 week (Figure 13(b) at 1 week: 58.2 ± 3.39 cells/mm^2^ in sham; 124.9 ± 5.54 cells/mm^2^ in ipsilateral SGZ; 97.5 ± 5.36 cells/mm^2^ in contralateral SGZ, *P* < 0.01 versus sham) and then decreased and stayed lower than sham significantly at 6 through 30 weeks after MCAO (Figure 13(b) at 30 weeks: 14.4 ± 2.21 cells/mm^2^ in ipsilateral MCAO; 14.8 ± 1.30 cells/mm^2^ in contralateral MCAO, *P* < 0.01 versus sham). Chronic IMP treatment significantly prevented the decreased DCX immunoreactivity at 30 weeks in MCAO groups, although they were not normalized as sham group (Figure 13(c): 26.2 ± 1.83 cells/mm^2^ in ipsilateral MCAO + IMP; 29.1 ± 3.84 cells/mm^2^ in contralateral MCAO + IMP; *P* < 0.05 versus its corresponding MCAO).

## 4. Discussion

Here we characterized a possible animal model of the postischemic depression after the transient MCAO in rats with a behavioral and an immunohistochemical analysis. Our findings in this study are as follows: (i) we observed spontaneous aggravation of anhedonia in MCAO-operated rats at chronic stages (20 and 30 weeks) after MCAO, which was prevented by chronic IMP or FLV treatment; (ii) this aggravation of anhedonia associated with delayed neuronal death of the granular neurons specifically in the ipsilateral DG, suggesting it would be a part of cellular and molecular pathophysiological mechanisms; (iii) the increased proliferation of NSCs and the following neural development in SGZ at early stages after MCAO were decreased significantly at chronic stages with almost ceased NSCs proliferation; (iv) the neurodegenerative changes in DG were prevented by chronic IMP treatment.

In the following, we discuss these subjects in further detail.

### 4.1. Postischemic Anhedonia in MCAO Rodents

From many previous studies combining MCAO and additional stressor in rodents [14], it is strongly suggested that the stress vulnerability after stroke is increased significantly. Without adding any contemplated stress, Boyko et al. reported % sucrose preference in permanent MCAO rats (operated on at 20 weeks of age and examined at 3 weeks after MCAO) decreased to around 60%, together with the increased immobility in FST and decreased responses in the shuttle avoidance task [39]. Craft and DeVries reported that the transient ischemia model of mice showed anhedonia with around 50% sucrose preference at 7 days after the surgical operation and was rescued by administration of an antagonist of interleukin-1 (IL-1) receptor [37]. Although both of them did not examine the time course change of anhedonia nor effect of antidepressants on it, these reports with our results in Figures 1(a) and 1(b) showed that PSD in rodents could be induced after MCAO without combining extra stressor stimuli. Sensitivity of the experimental anhedonia to antidepressants should carefully be examined because as shown in Figures 1(a) and 1(b), lessor sucrose preference in MCAO rats was observed throughout the time course from 1 day to 6 weeks after MCAO, and it was insensitive to IMP or FLV. It should also be noted that the decreased amount of daily water consumption in MCAO rats was not rescued by IMP nor by FLV, suggesting that some hypothalamic functions controlling water intake may be affected after MCAO. Because of this reason, we had to increase the concentration of antidepressants in the drinking water of MCAO rats so that they take the same amount of antidepressants as sham rats. To avoid this issue, use of an osmotic minipump, for example, would be examined in further studies.

### 4.2. Postischemic Neuronal Loss and Apoptosis in GCL

As mentioned in the Introduction, delayed neuronal death of the hippocampal CA1 pyramidal neurons after ischemia has been well characterized, and other regions such as CA3 and DG were thought to be resistant to ischemic stress. In this study, however, we demonstrated delayed neuronal death of the granular neurons in DG at chronic stages after MCAO for the first time, which was consistent with the time course of the aggravation of anhedonia (Figures 1(a), 2–6). Considering the increased immunoreactivities of cleaved caspase-3 and Bax together with decreased Bcl-2*α* at chronic stages in the ipsilateral GCL (Figures 7–9), it seems that the death signal exceeded the survival signal in GCL after 1 week of MCAO (Figure 10), resulting in the increased apoptotic neuronal death of the matured GC. The increased Bax after 1 week stayed higher than that of sham, whereas Bcl-2*α* significantly decreased compared to sham in those periods, suggesting that the death signal exceeded the survival signal. Further studies are required to identify what kind of molecular factor triggers this neurodegenerative condition. One possible mechanism is the increase of the inflammatory factors such as tumor-necrosis factor *α* (TNF*α*), IL-1*β*, IL-6, and iNOS from glial cells [22, 40, 41]. In fact, trimethyltin was reported to induce selective loss of granular neurons in DG with activating TNF*α*-mediated apoptotic signaling [42, 43]. Other possible molecular candidates are the decreased trophic factors such as brain-derived neurotrophic factor (BDNF), nerve growth factor (NGF) [44, 45], and vascular endothelial growth factor (VEGF) [46]. BDNF expression in PSD animals has been examined by researchers [14, 18, 39]. Considering the neuronal activity-dependent production of neurotrophic factors [47], it is also possible that the brain areas innervating DG such as the entorhinal cortex might be damaged after MCAO with decreasing the neuronal input to DG; thus it reduced the neuronal activities of DG, resulting in the loss of neurotrophic conditions. Further electrophysiological, neurochemical, and immunohistochemical examinations will explore the detailed pathological mechanisms of the neurodegeneration in the ipsilateral DG after MCAO.

### 4.3. Postischemic Proliferation of the Neural Stem Cells in SGZ

Increased proliferation of NSCs after ischemic event in SGZ was reported previously [25, 26]. It was also indicated that the proliferated NSCs differentiated into mature neurons and integrated in GCL [48]. In fact, our results of Nestin, Ki67, and DCX immunostaining at early stages after MCAO (Figures 11–13) were consistent with these reports. Although this initial increase is thought to be important to keep the number of mature GC after MCAO for at least 20 weeks, it did not last for 2 weeks and dropped under the sham levels after 6 weeks of MCAO in our observations (Figures 11–13). It seemed that NSCs in SGZ after 6 weeks of MCAO escaped from cell cycle and stayed quiescent. The decreased apoptosis in SGZ after 20 weeks of MCAO also supports this idea (Figures 7 and 8). Different from the loss of GC specifically observed in the ipsilateral GCL, time course changes of NSCs were observed in both ipsi- and contralateral sides of SGZ, suggesting that they were controlled by systemic regulatory mechanisms such as glucocorticoids released according to the activation of hypothalamic-pituitary-adrenal (HPA) axis [49]. GC with condensed cytosol stained by cresyl violet also tended to increase in both ipsi- and contralateral DG (see Section 3.3, 2nd paragraph). Together with the apoptotic loss of the ipsilateral GC, the suppressed neurogenesis of the bilateral SGZ would also contribute to the aggravation of anhedonia as a result of response to the psychogenic stressor triggered by such motor impairments.

### 4.4. Effects of Antidepressants after MCAO

Because both IMP and FLV prevented aggravation of anhedonia after 20 weeks of MCAO (Figure 1), we confirmed it was closely related to PSD. FLV is a SSRI, which primarily increases serotonin and dopamine together with stimulating sigma-1 receptor [50]. Recent study of Zhang et al. showed decreased sucrose preference, increased immobility in forced swim and tail suspension tests, and exacerbated neurological functions with reduced body weight by combining MCAO and spatial restraint stress in mice [18]. They also showed the decreased serotonin and dopamine levels in hypothalamus, hippocampus, and cortex in their PSD mice, which were reversed by chronic IMP treatment. Thus, PSD is thought to imply decreased serotonin and dopamine levels in brain with increased cortisol level in serum.

Based on the results of Bax and Bcl-2*α* analysis shown in Figures 8 and 9, chronic IMP treatment seemed to stimulate the survival signal rather than suppressing the death signal in GCL. Neuroprotective effect of antidepressants was suggested to mediate BDNF-cyclic AMP responsive element-binding (CREB) protein pathway in a previous report [51], and this could be one possible molecular mechanism to keep GC alive by antidepressants after MCAO. Antidepressants including IMP and FLV are also known to stimulate proliferation of NSCs in SGZ, thus promoting the increase of adult neurogenesis [27]. It is understood to be one of the essential mechanisms for the effects of antidepressants [52]. In fact, our results in Figures 11–13 showed that IMP tended to stimulate the proliferation of NSCs, although it did not normalize to the sham level in MCAO groups in our experimental condition. Considering our time course data of both apoptosis and neurogenesis with the effect of antidepressants, favorable timing for the onset of antidepressant treatment on PSD seems to be within 2 weeks after MCAO, although it should be carefully examined with considering the species difference between rodents and humans. Moreover, effects of antidepressants after establishing anhedonia should be examined in our future study, because it will clarify whether antidepressants have therapeutic potency in addition to neuroprotective effects in our PSD model.

## 5. Conclusions

Spontaneous aggravation of anhedonia was observed at the chronic stage in a rat model of transient cerebral focal ischemia. It associated with the neurodegenerative changes in the hippocampal dentate gyrus: apoptotic neuronal loss of the granular cells and decreased proliferation of the neural stem cells. They are prevented by chronic administration of antidepressants. Our study provides new insights into the pathophysiological mechanisms of poststroke depression and effective use of antidepressants on it.

## Supplementary Material

The supplemental figures show Bax and Bcl-2α immunoreactivities in the contralateral GCL and SGZ. See Section 3.5 for details.

## Abbreviations

ANOVA: Analysis of variance
BDNF: Brain-derived neurotrophic factor
CNS: Central nervous system
CREB protein: Cyclic AMP responsive element-binding protein
DAB: 3′,3′-Diaminobenzidine
DCX: Doublecortin
DG: Dentate gyrus
FLV: Fluvoxamine
GCL: Granular cell layer
HPA axis: Hypothalamus-pituitary-adrenal axis
IL: Interleukin
IMP: Imipramine
MCAO: Middle cerebral artery occlusion
NGF: Nerve growth factor
NSCs: Neural stem cells
PSD: Poststroke depression
SGZ: Subgranular zone
SSRI: Selective serotonin reuptake inhibitor
SVZ: Subventricular zone
TNF*α*: Tumor-necrosis factor *α*
UCMS: Unpredicted chronic mild stress
VEGF: Vascular endothelial growth factor.
